# Supplementary material for: Molecular analysis for ovarian cancer detection in patient-friendly samples
Source: Commun Med (Lond). 2024 May 16;4:88. doi: 10.1038/s43856-024-00517-8 (PMC11099128; doi:10.1038/s43856-024-00517-8)
Supplement: Supplementary file 1 — Supplementary Information [file 43856_2024_517_MOESM1_ESM.pdf]

Supplemental Information for:

Molecular analysis for ovarian cancer detection in patient-friendly samples

Birgit M.M. Wever[1,2], Mirte Schaafsma[1,2,3], Maaike C.G. Bleeker[1,2], Yara van den Burgt[1,2], Rianne van den Helder[1,2,3], Christianne A.R. Lok[3], Frederike Dijk[2,4], Ymke van der Pol[1,2], Florent Mouliere[1,2], Norbert Moldovan[1,2], Nienke E. van Trommel[3], Renske D.M. Steenbergen[1,2*]

[1] Amsterdam UMC, location Vrije Universiteit Amsterdam, Department of Pathology, Amsterdam, The Netherlands

[2] Cancer Center Amsterdam, Imaging and Biomarkers, Amsterdam, The Netherlands

[3] Antoni van Leeuwenhoek/Netherlands Cancer Institute, Department of Gynecologic Oncology, Center of Gynecologic Oncology Amsterdam, Amsterdam, The Netherlands

[4] Amsterdam UMC, location University of Amsterdam, Department of Pathology, Amsterdam, The Netherlands

[*]**Corresponding author:** Amsterdam UMC, Vrije Universiteit Amsterdam, Department of Pathology, Cancer Center Amsterdam, De Boelelaan 1117, PO Box 7057, 1007 MB, Amsterdam, The Netherlands. Telephone: 00-31-204442331; Fax: 00-31-204442964; E-mail: r.steenbergen@amsterdamumc.nl

SUPPLEMENTAL METHODS

Sample collection and processing

Urine and cervicovaginal self-samples were collected at home for which all participants received a package including materials needed for collection and transport. Participants were instructed to collect urine before the cervicovaginal self-sample. Cervicovaginal self-samples were collected according to the provided user manual using the Evalyn® brush (Rovers Medical Devices, Oss, The Netherlands), which is a clinically validated self-sampling method[1]. Urine was collected in 3x30 mL tubes containing the storage buffer Ethylenediaminetetraacetic acid (EDTA; final concentration 40mM) to preserve nucleic acids during transport. Clinician-taken cervical scrapes were collected prior to surgery using a Cervex-Brush (Rovers Medical Devices) and directly placed in 10 mL Thinprep PreservCyt medium (Hologic, Marlborough, MA, US). Samples were sent to the Pathology department of Amsterdam UMC, location VUmc, within 72 hours by regular mail and processed directly after arrival.

Urine was processed as described in our previously validated processing and storage protocol[2]. Briefly, a total of 15 mL of urine was centrifuged at 3000g for 10 min to separate the urine into two fractions: the urine supernatant and urine sediment. Both fractions and the remaining full void (*i.e.* unfractionated) urine were stored at -20°C. Cytological samples were processed as described previously for cervical[3] and endometrial cancer[4]. Cervicovaginal self-samples were stored in 1.5 mL ThinPrep PreservCyt medium upon arrival. Cervicovaginal self-samples and cervical scrapes were stored at 4°C.

Formalin-fixed paraffin-embedded (FFPE) and fresh frozen tissue specimens were consecutively sectioned of which the first and last sections were Hematoxylin and Eosin (H&E) stained for histopathological review by a pathologist to confirm the presence of ovarian cancer or normal fallopian tube tissue.

DNA extraction and bisulfite modification

DNA from full void urine (30 mL patients diagnosed with ovarian mass; 40 mL controls), urine sediment (15 mL original volume), and urine supernatant (15 mL) was extracted as described previously[5,6]. In short, both full void urine and urine supernatant were isolated with the Quick DNA urine kit (Zymo Research, Irvine, CA, US) and urine sediment using the DNA mini and blood mini kit (Qiagen, Hilden,

Germany). DNA from cervicovaginal self-samples and clinician-taken cervical scrapes was isolated as described before[3], using the NucleoMag 96 Tissue kit (Machery-Nagel) and a Microlab Star robotic system (Hamilton, Germany). DNA of FFPE tissue samples was isolated using the QIAamp DNA FFPE tissue kit (Qiagen, Hilden, Germany). DNA of fresh frozen tissue samples was isolated using the DNeasy Blood & Tissue kit (Qiagen). DNA yield was quantified using a NanoDrop 1000 (Thermo Fisher Scientific, Waltham, MA, US). Up to 250 ng of extracted DNA was subjected to bisulfite modification using the EZ DNA Methylation Kit (Zymo Research) to convert unmethylated cytosines. All procedures were performed according to manufacturers' guidelines.

Reaction conditions and instrument identifications of quantitative methylation-specific PCR

Up to 50 ng of modified DNA was mixed with Epitect Multiplex PCR Mastermix (Qiagen, Venlo, Netherlands), 2.5-5.0 µM of each primer, and 5.0-10.0 µM of each hydrolysis probe in a total volume of 12.5 µl. Thermocycling conditions were: 95°C for 5 minutes, 45 cycles at 95°C for 15 seconds, 59/60/63°C for 1 minute, and 72°C for 1 minute. Quantitative methylation-specific PCR (qMSP) assays were performed using a ViiA7 real-time PCR-system (Applied Biosystems, Foster City, CA, USA) or an ABI-7500 real-time PCR-system (Applied Biosystems, Waltham, MA, US) for *GHSR/SST/ZIC1*. The qMSP data was analyzed with manual thresholds and automatic baseline settings using QuantStudio™ Real-Time PCR Software (v. 1.6.1) and 7500 Software (v. 2.3).

Analysis of somatic copy number aberrations and cell-free DNA fragmentation patterns

Processing of the sequencing data was performed by a pipeline controlled by Snakemake (v. 7.14.0). In brief, sequencing adapters and indexes were trimmed by the bbduk.sh (v. 38.79) [https://sourceforge.net/projects/bbmap/] in paired mode with parameters 'ktrim=r k=23 mink=11 hdist=1' and the adapter reference dataset provided with the software. Trimmed non-converted samples were mapped to the GRCh38 human genome assembly (GeneBank accession: GCA_000001405.28) using bwa mem (v. 0.7.17) [https://github.com/lh3/bwa]. Enzymatically converted reads were mapped to the same assembly using biscuit (v. 1.0.2.20220113) [https://huishenlab.github.io/biscuit/]. For both non-converted and converted samples, reads with a mapping quality lower than 5, unmapped reads, secondary mappings, chimeric and PCR duplicates were filtered using samtools (v. 1.12) [https://github.com/samtools/samtools] and sambamba markdup (v. 0.8.1) [https://lomereiter.github.io/sambamba/]. Reads passing the filtering step were submitted for somatic

copy number aberrations (SCNA) analysis and tumor fraction estimation using the ichorCNA software (v. 0.3.2.0)[7] using default settings, except the use of an in-house panel-of-normals from shallow whole-genome sequencing, setting the non-tumor fraction parameter restart values to c(0.95,0.99,0.995,0.999). The tumor fraction with the highest log likelihood was reported. Fragmentation patterns of urine cfDNA for both non-converted and converted samples were analyzed by retrieving the fragment sizes of the trimmed and filtered reads using picard CollectInsertSizeMetrics (v. 2.22.2) with HISTOGRAM_WIDTH=1000 [https://gatk.broadinstitute.org/hc/en-us].

Shallow whole-genome sequencing for the analysis of SCNA in paired FFPE primary tumor tissue was performed as described previously with a few adaptations[8]. Sequencing libraries were prepared using the KAPA HyperPlus Kit (Roche, Basel, Switzerland), following manufacturer's protocol. Libraries were sequenced using a NextSeq2000 (Illumina). Sequence reads were aligned to the GRCh38 human genome assembly using bwa mem (v. 0.7.17). PCR duplicates (marked by Picard v. 2.20.8), as well as low-quality reads (MAPQ < 37), were filtered out using samtools (v. 0.1.1830). Reads passing the filtering step were submitted for SCNA analysis using ichorCNA software as described for urine samples.

SUPPLEMENTAL REFERENCES

1 Bosgraaf, R. P. *et al.* Comparative performance of novel self-sampling methods in detecting high-risk human papillomavirus in 30,130 women not attending cervical screening. *Int J Cancer* **136**, 646-655 (2015). https://doi.org:10.1002/ijc.29026

2 Bosschieter, J. *et al.* A protocol for urine collection and storage prior to DNA methylation analysis. *PLoS One* **13**, e0200906 (2018). https://doi.org:10.1371/journal.pone.0200906

3 van den Helder, R. *et al.* HPV and DNA Methylation Testing in Urine for Cervical Intraepithelial Neoplasia and Cervical Cancer Detection. *Clinical Cancer Research* **28**, 2061-2068 (2022). https://doi.org:10.1158/1078-0432.Ccr-21-3710

4 Wever, B. M. M. *et al.* DNA methylation testing for endometrial cancer detection in urine, cervicovaginal self-samples, and cervical scrapes. *Int J Cancer* (2023). https://doi.org:10.1002/ijc.34504

5 van den Helder, R. *et al.* Non-invasive detection of endometrial cancer by DNA methylation analysis in urine. *Clinical Epigenetics* **12**, 165 (2020). https://doi.org:10.1186/s13148-020-00958-7

6 van den Helder, R., van Trommel, N. E., van Splunter, A. P., Lissenberg-Witte, B. I., Bleeker, M. C. G. & Steenbergen, R. D. M. Methylation analysis in urine fractions for optimal CIN3 and cervical cancer detection. *Papillomavirus Res* **9**, 100193 (2020). https://doi.org:10.1016/j.pvr.2020.100193

7 Adalsteinsson, V. A. *et al.* Scalable whole-exome sequencing of cell-free DNA reveals high concordance with metastatic tumors. *Nature Communications* **8**, 1324 (2017). https://doi.org:10.1038/s41467-017-00965-y

8 Scheinin, I. *et al.* DNA copy number analysis of fresh and formalin-fixed specimens by shallow whole-genome sequencing with identification and exclusion of problematic regions in the genome assembly. *Genome Res* **24**, 2022-2032 (2014). https://doi.org:10.1101/gr.175141.114

SUPPLEMENTAL FIGURES

Table of contents

Supplemental Figure 1
DNA methylation levels of *C2CD4D, CDO1, GALR1, GHSR, MAL, NRN1, PRDM14, SST,* and *ZIC1* in high grade serous ovarian cancer (n=35) and normal fallopian tube tissue (n=22).

Supplemental Figure 2
DNA methylation levels of *C2CD4D, CDO1, GALR1, GHSR, MAL, NRN1, PRDM14, SST,* and *ZIC1* in full void (*i.e.* unfractionated) urine of healthy controls (n=30), and women diagnosed with a benign (n=27) or high stage malignant ovarian mass (n=28).

Supplemental Figure 3
DNA methylation levels of *C2CD4D, CDO1, GALR1, GHSR, MAL, NRN1, PRDM14, SST,* and *ZIC1* in urine supernatant of healthy controls (n=29), and women diagnosed with a benign (n=27) or high stage malignant ovarian mass (n=29).

Supplemental Figure 4
DNA methylation levels of *C2CD4D, CDO1, GALR1, GHSR, MAL, NRN1, PRDM14, SST,* and *ZIC1* in urine sediment of healthy controls (n=30), and women diagnosed with a benign (n=27) or high stage malignant ovarian mass (n=29).

Supplemental Figure 5
DNA methylation levels of *C2CD4D, CDO1, GALR1, GHSR, MAL, NRN1, PRDM14, SST,* and *ZIC1* in clinician-collected cervical scrapes of healthy controls (n=40), and women diagnosed with a benign (n=23) or high stage malignant ovarian mass (n=24).

Supplemental Figure 6
DNA methylation levels of *C2CD4D, CDO1, GALR1, GHSR, MAL, NRN1, PRDM14, SST,* and *ZIC1* in self-collected cervicovaginal samples of healthy controls (n=40), and women diagnosed with a benign (n=25) or high stage malignant ovarian mass (n=28).

Supplemental Figure 7
Boxplots visualizing *ACTB* Cq levels per multiplex (1: *GHSR/SST/ZIC1*, 2: *CDO1/MAL/PRDM14*, 3: *C2CD4D/GALR1/NRN1*).

Supplemental Figure 8
Boxplots illustrating an additional analysis of methylation levels of most discriminating markers using a lower *ACTB* Cq threshold.

Supplemental Figure 9
The Spearman correlation coefficients (*r*) of methylation markers *C2CD4D, CDO1, GHSR,* and *MAL* between paired samples of 23 women diagnosed with ovarian cancer.

Supplemental Figure 10
Genome-wide SCNA profiles of matched urine and FFPE primary tumor tissue.

Supplemental Figure 11
Scatter plot indicating the relation between *MAL* methylation levels and the tumor fraction as estimated by ichorCNA in urine supernatant samples.

Supplemental Figure 12
Fragment size distributions for cfDNA reads of urine supernatant samples from healthy controls (n=2) and ovarian cancer patients with a low (<5%, n=19) and high (≥5%, n=4) tumor fraction determined from shallow whole-genome sequencing.

Supplemental Figure 1: DNA methylation levels of *C2CD4D, CDO1, GALR1, GHSR, MAL, NRN1, PRDM14, SST,* and *ZIC1* in high grade serous ovarian cancer (n=35) and normal fallopian tube tissue (n=22). DNA methylation levels are shown by 2log-transformed Cq ratios. Violin plots represent medians with lower and upper quartile and range whiskers. A *p*-value of 0.05 was considered statistically significant. ****: $p < 0.0001$. Cq = quantification cycle; HGSOC = high grade serous ovarian cancer.

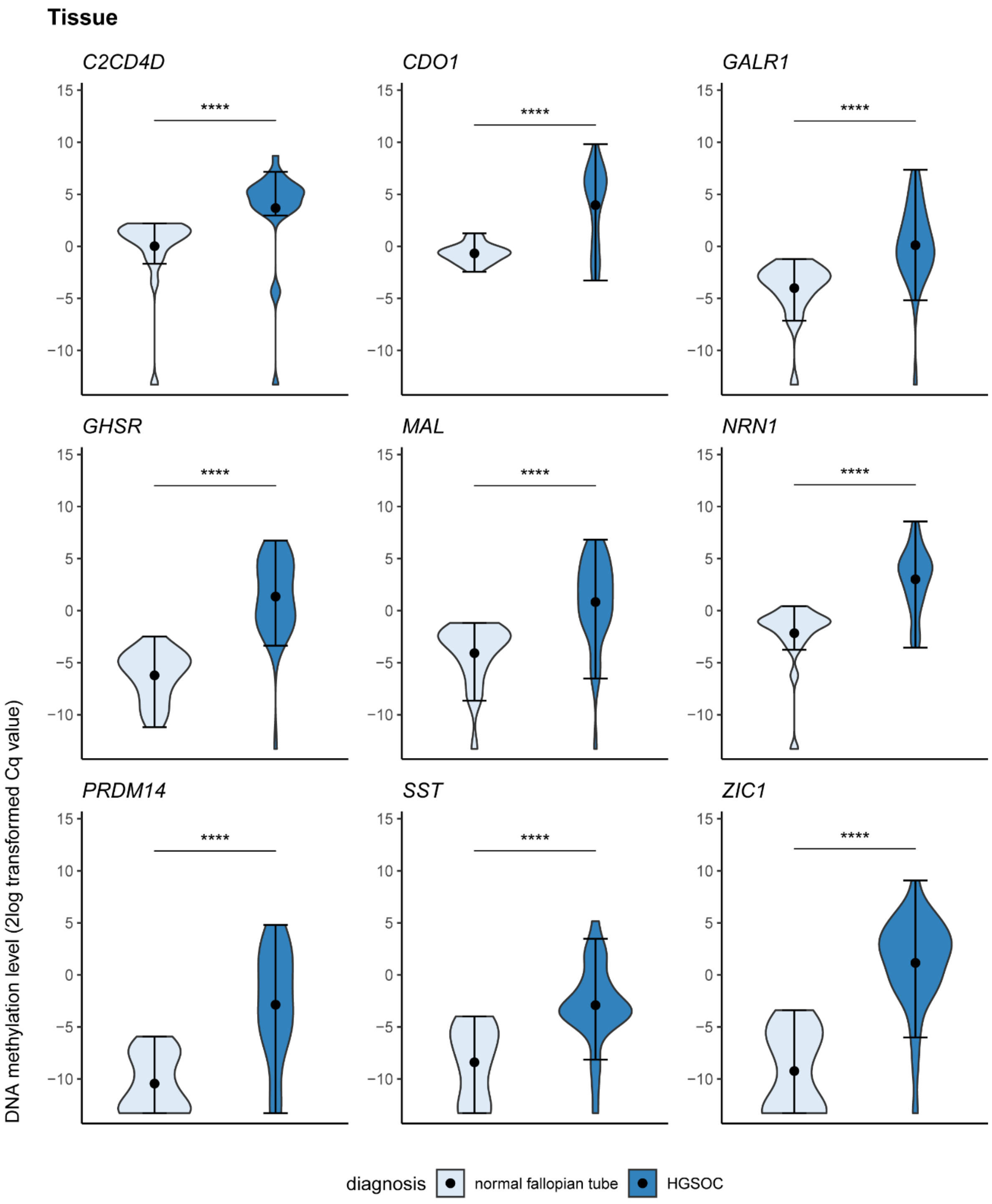

Supplemental Figure 2: DNA methylation levels of *C2CD4D, CDO1, GALR1, GHSR, MAL, NRN1, PRDM14, SST,* and *ZIC1* in full void (*i.e.* unfractionated) urine of healthy controls (n=30), and women diagnosed with a benign (n=27) or high stage malignant ovarian mass (n=28). DNA methylation levels are shown by 2log-transformed Cq ratios. Violin plots represent medians with lower and upper quartile and range whiskers. A *p*-value of <0.05 was considered statistically significant. Cq = quantification cycle.

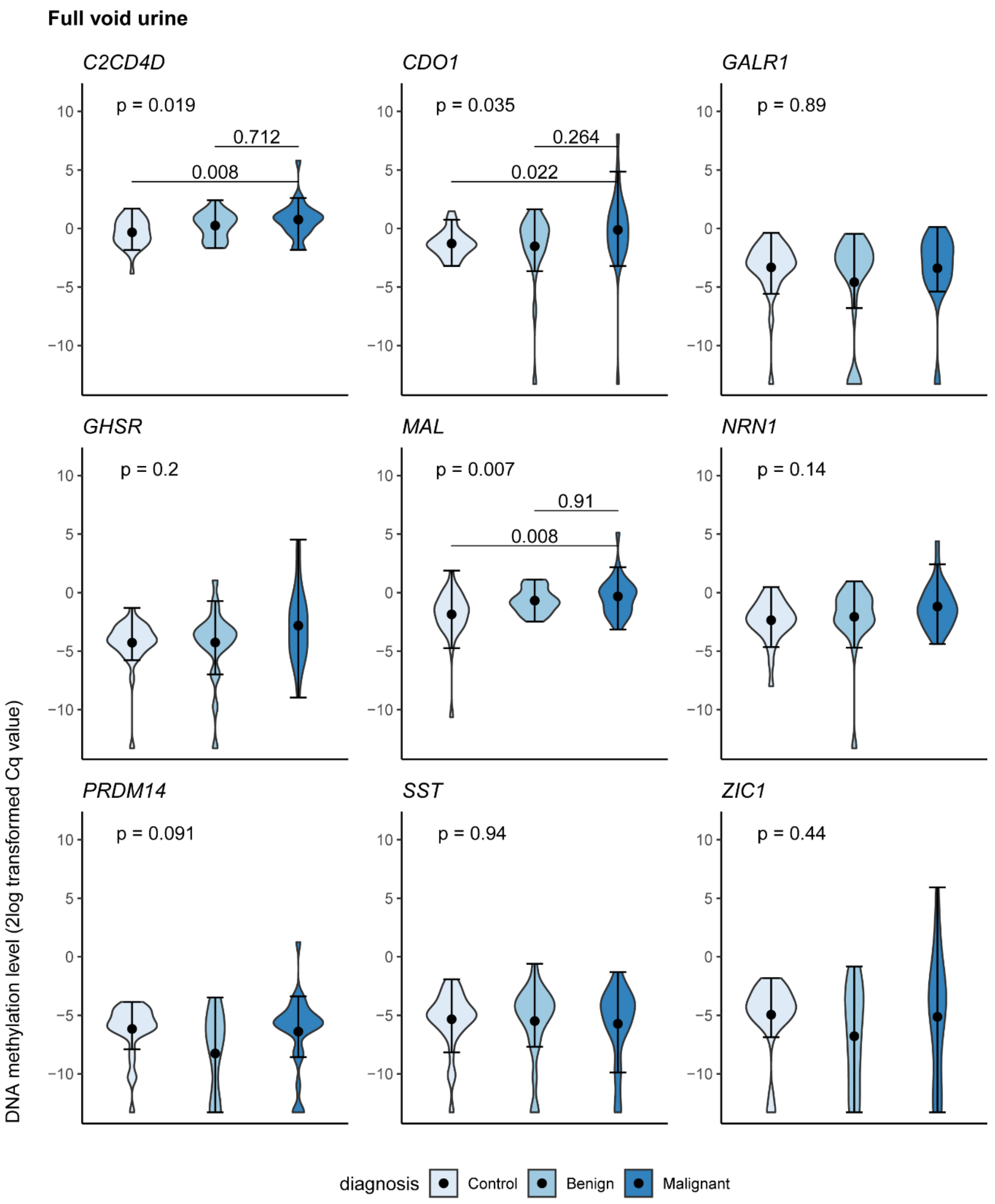

Supplemental Figure 3: DNA methylation levels of *C2CD4D, CDO1, GALR1, GHSR, MAL, NRN1, PRDM14, SST,* and *ZIC1* in urine supernatant of healthy controls (n=29), and women diagnosed with a benign (n=27) or high stage malignant ovarian mass (n=29). DNA methylation levels are shown by 2log-transformed Cq ratios. Violin plots represent medians with lower and upper quartile and range whiskers. A *p*-value of <0.05 was considered statistically significant. Cq = quantification cycle.

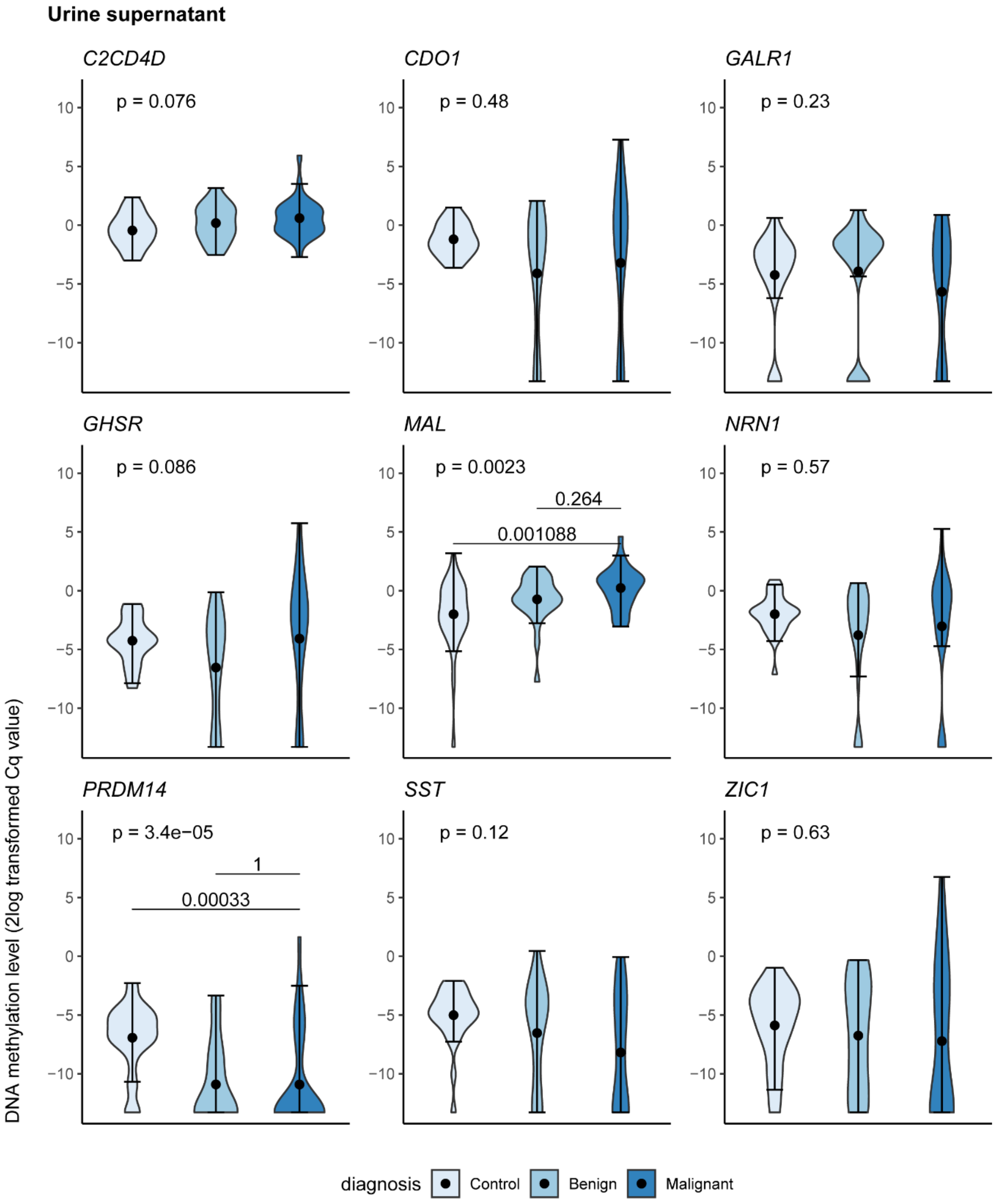

Supplemental Figure 4: DNA methylation levels of *C2CD4D, CDO1, GALR1, GHSR, MAL, NRN1, PRDM14, SST,* and *ZIC1* in urine sediment of healthy controls (n=30), and women diagnosed with a benign (n=27) or high stage malignant ovarian mass (n=29). DNA methylation levels are shown by 2log-transformed Cq ratios. Violin plots represent medians with lower and upper quartile and range whiskers. A *p*-value of <0.05 was considered statistically significant. Cq = quantification cycle.

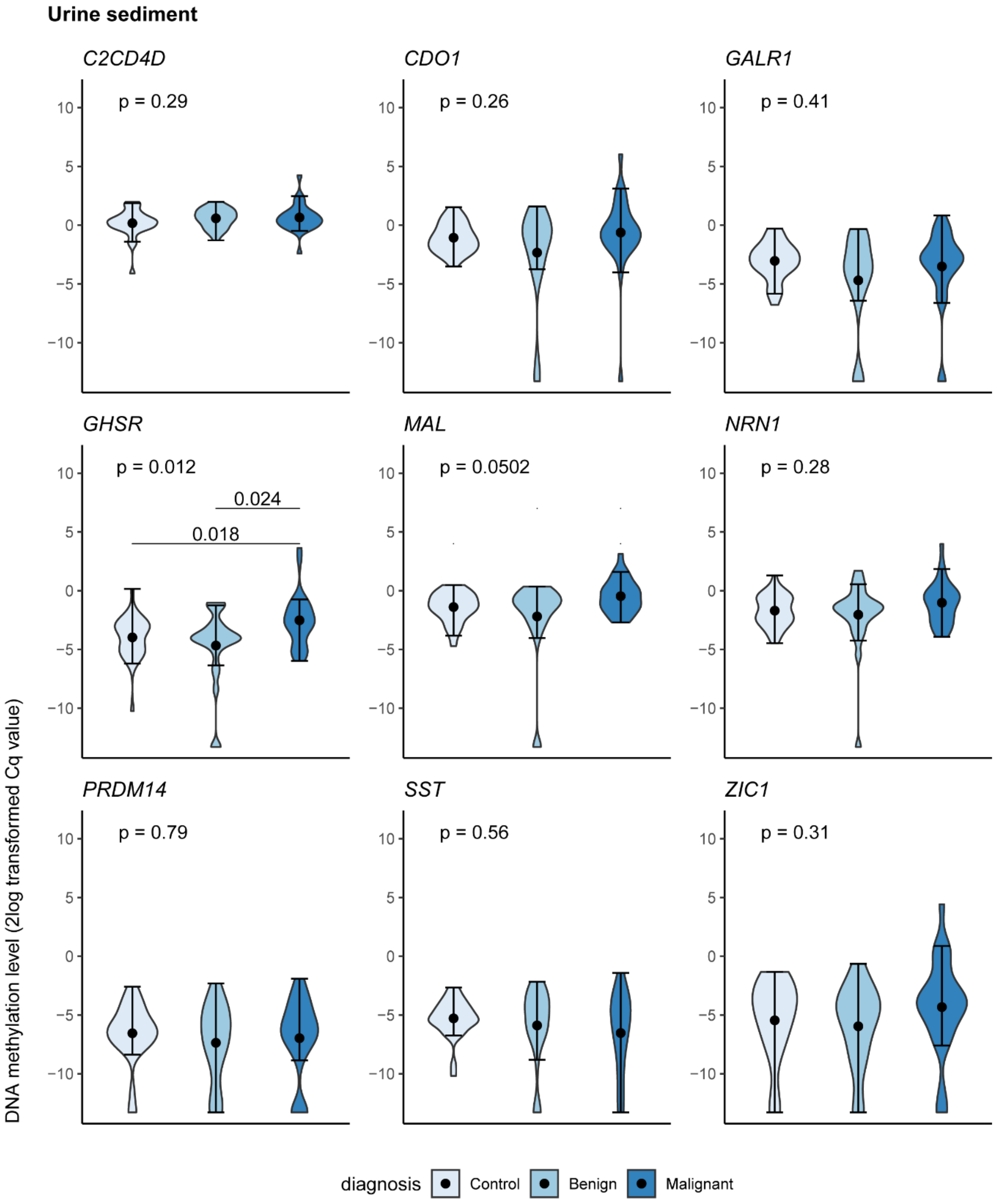

Supplemental Figure 5: DNA methylation levels of *C2CD4D, CDO1, GALR1, GHSR, MAL, NRN1, PRDM14, SST,* and *ZIC1* in clinician-taken cervical scrapes of healthy controls (n=40), and women diagnosed with a benign (n=23) or high stage malignant ovarian mass (n=24). DNA methylation levels are shown by 2log-transformed Cq ratios. Violin plots represent medians with lower and upper quartile and range whiskers. A *p*-value of <0.05 was considered statistically significant. Cq = quantification cycle.

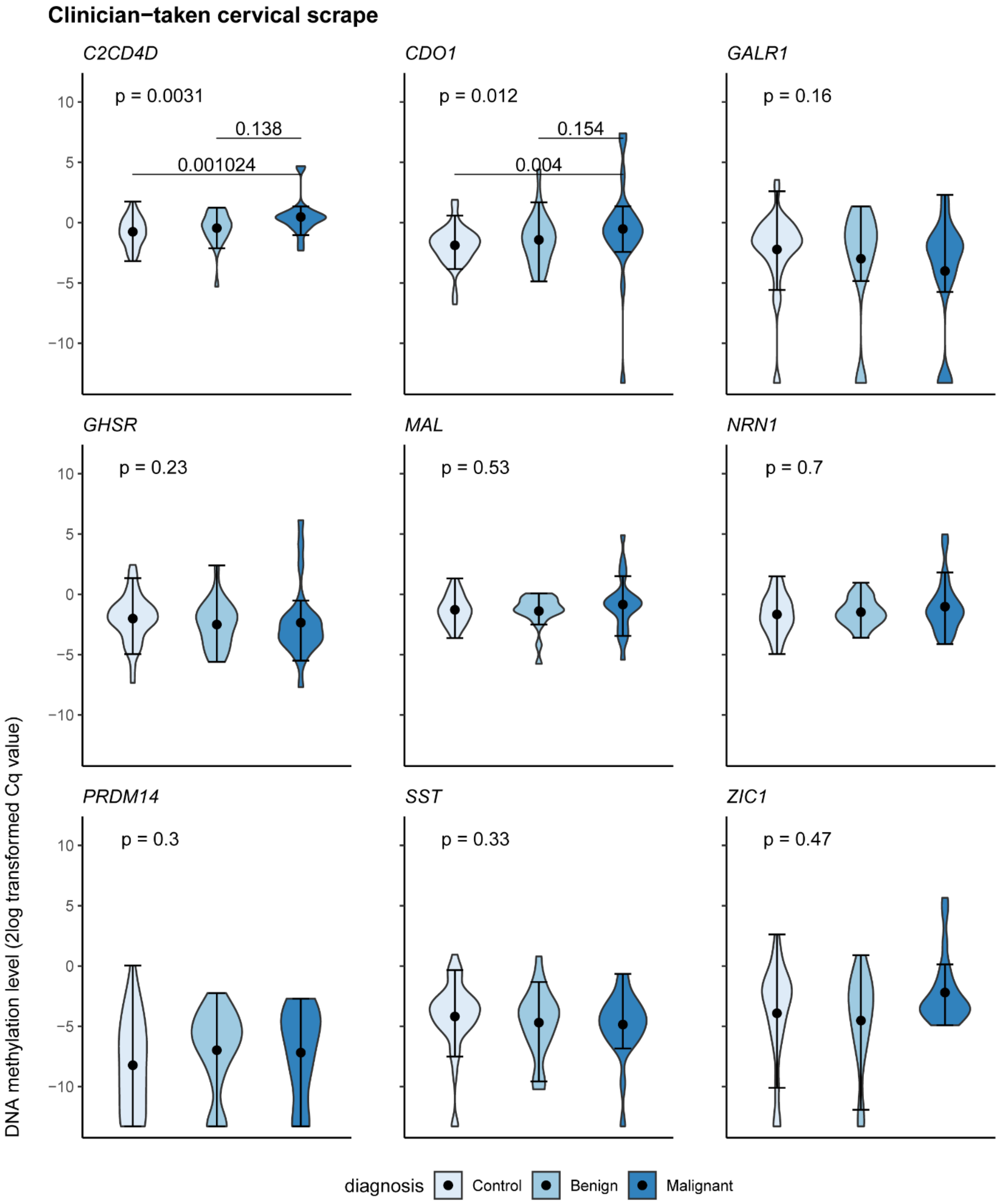

Supplemental Figure 6: DNA methylation levels of *C2CD4D, CDO1, GALR1, GHSR, MAL, NRN1, PRDM14, SST,* and *ZIC1* in self-collected cervicovaginal samples of healthy controls (n=40), and women diagnosed with a benign (n=25) or high stage malignant ovarian mass (n=28). Violin plots represent medians with lower and upper quartile and range whiskers. DNA methylation levels are shown by 2log-transformed Cq ratios. A *p*-value of <0.05 was considered statistically significant. Cq = quantification cycle.

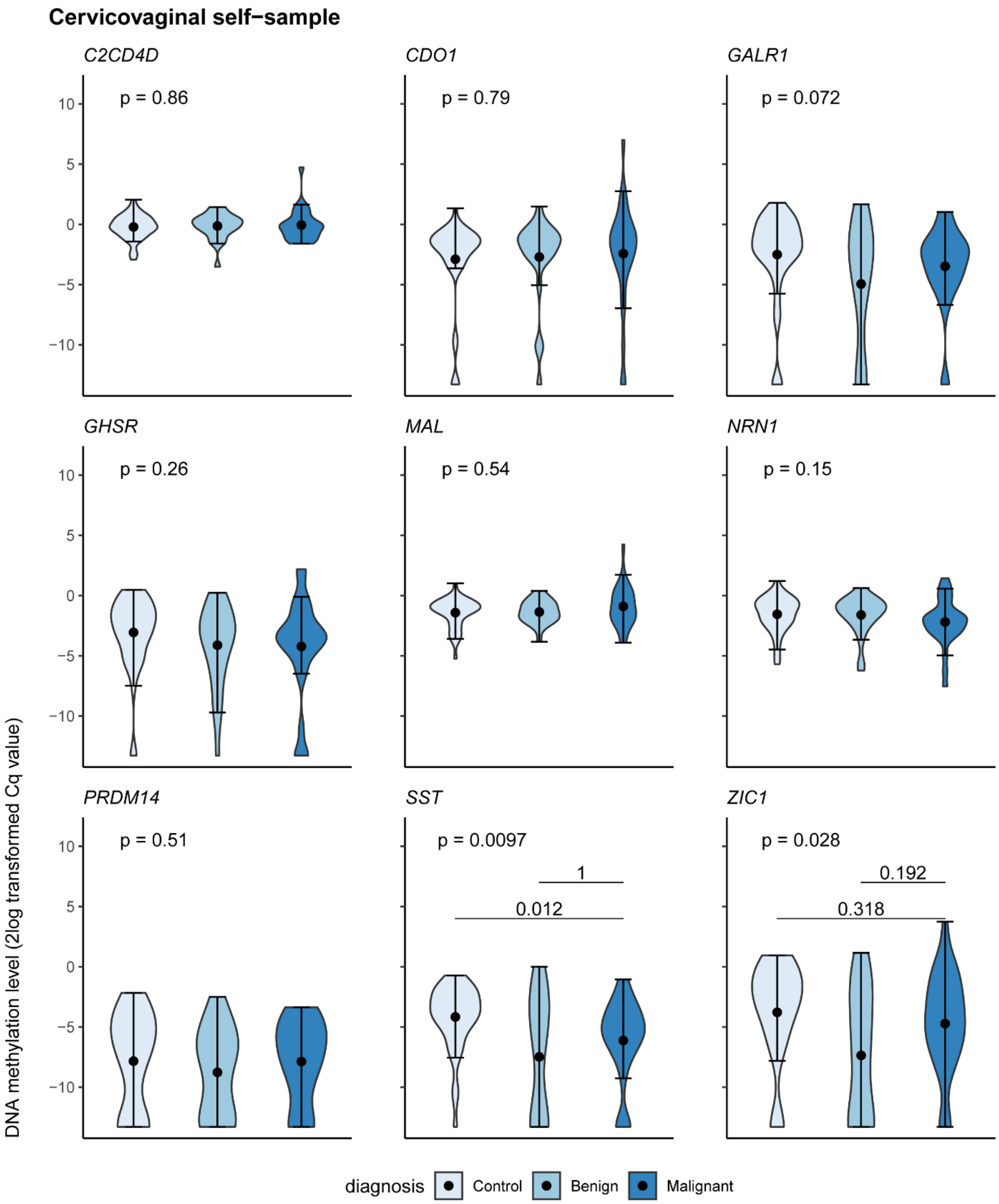

Supplemental Figure 7: Boxplots visualizing *ACTB* Cq levels per multiplex (1: *GHSR/SST/ZIC1*, 2: *CDO1/MAL/PRDM14*, 3: *C2CD4D/GALR1/NRN1*). *ACTB* is used as reference gene for methylation analysis and included in each multiplex. Sample quality and sufficient input was ensured by excluding samples with a *ACTB* Cq ≥ 32. An additional analysis was performed to explore the use of a lower *ACTB* threshold and to determine whether significant results remain. The *ACTB* Cq threshold of 30 (green line) was used for this additional analysis (Supplemental Figure 8). Groups are stratified per sample type and diagnostic category. Cq = quantification cycle.

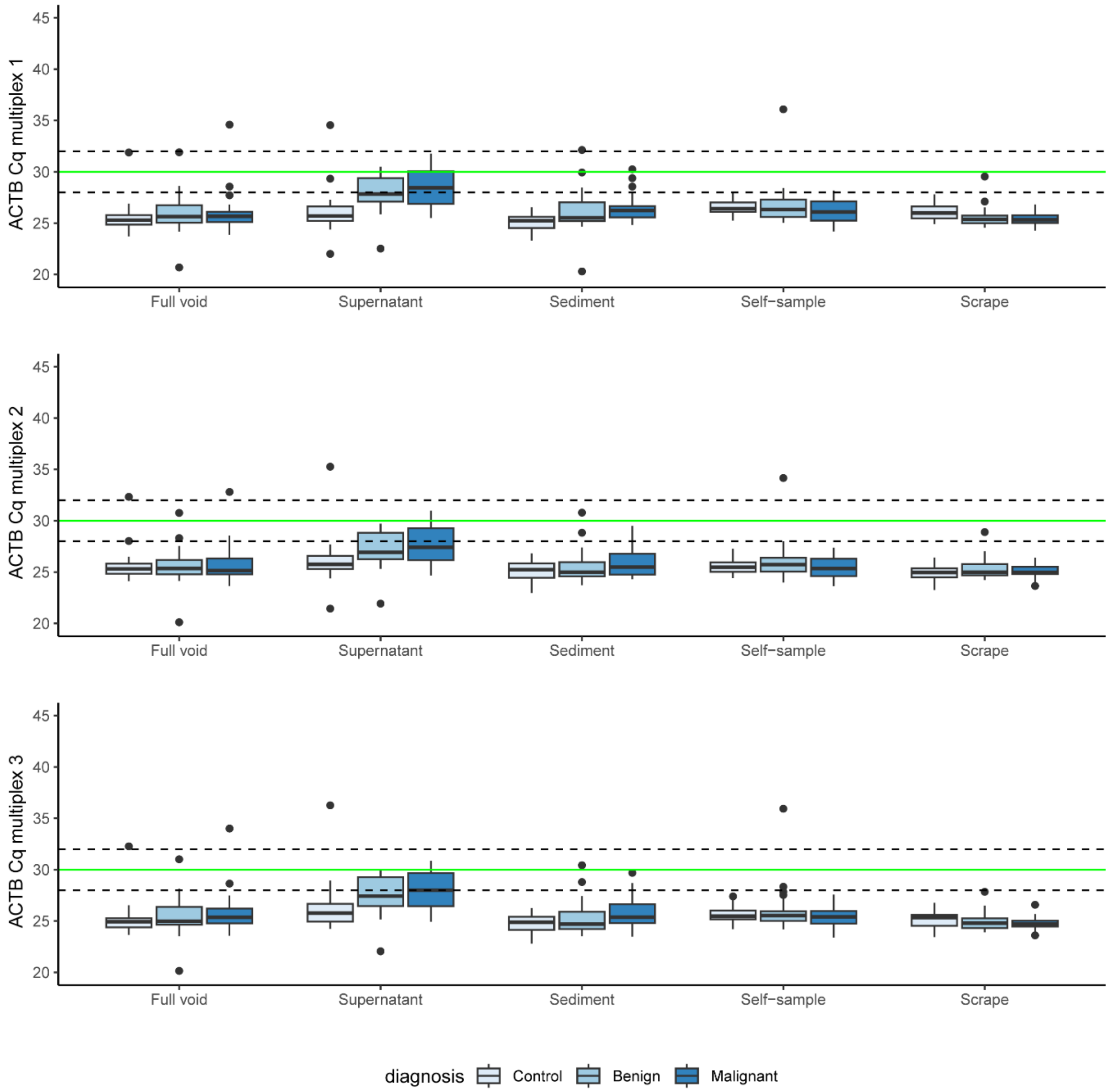

Supplemental Figure 8: Boxplots illustrating an additional analysis of methylation levels of most discriminating markers using a lower *ACTB* Cq threshold. Significant differences between the diagnostic categories remain when using a *ACTB* Cq threshold of ≥ 30, instead of ≥ 32. Methylation levels are expressed by 2log-transformed Cq ratios. Violin plots represent medians with lower and upper quartile and range whiskers. *P*-values shown are Bonferroni corrected (*i.e.* divided by the number of diagnostic groups compared) and considered statistically significant when <0.05.

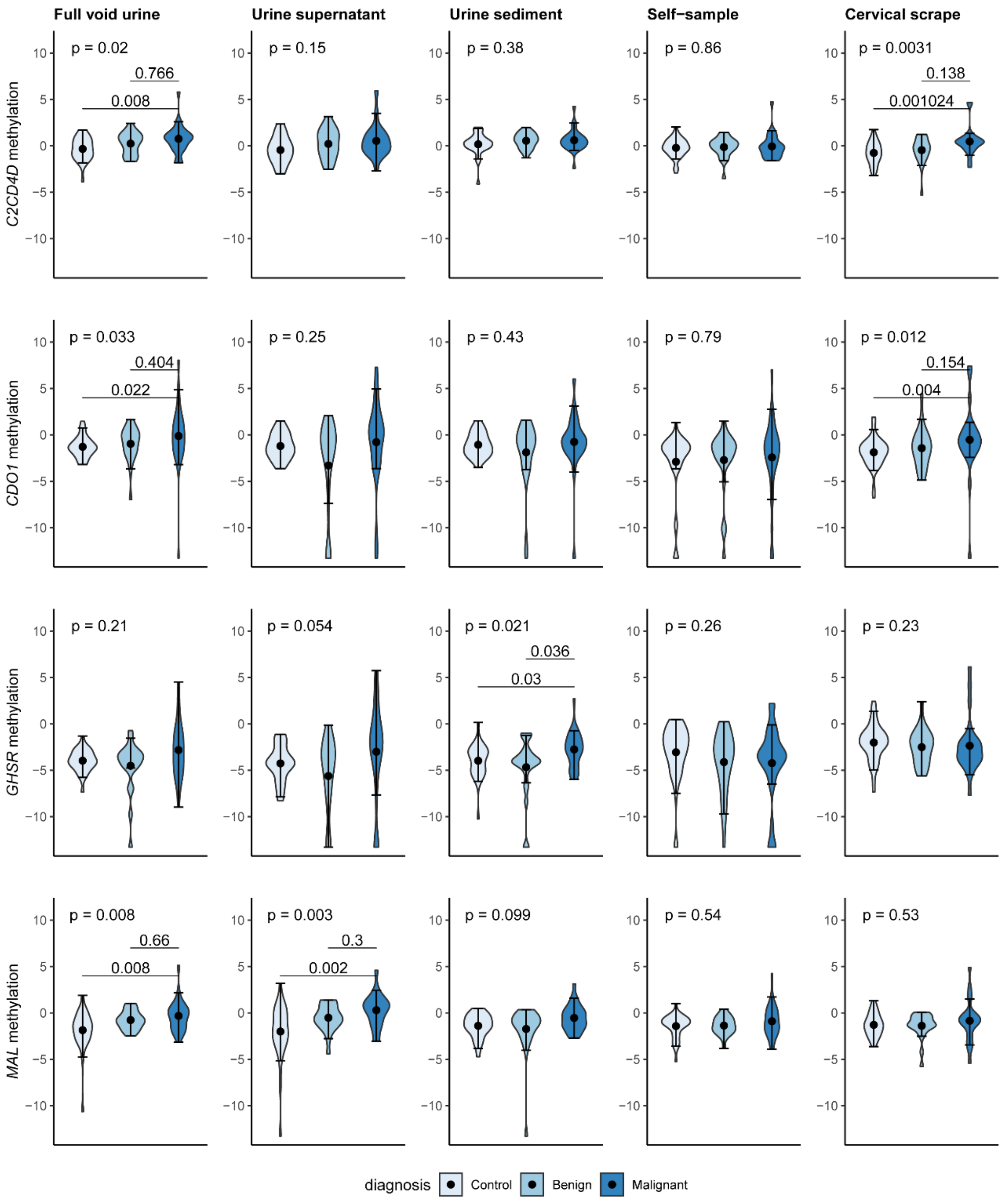

Supplemental Figure 9: The Spearman correlation coefficients (*r*) of methylation markers *C2CD4D, CDO1, GHSR,* and *MAL* between paired samples of 23 women diagnosed with ovarian cancer. The Spearman correlation coefficient was calculated based on 2log-transformed Cq ratios. Circle color and size indicate the degree of correlation (*i.e.*, the larger and darker the circle, the more correlation).

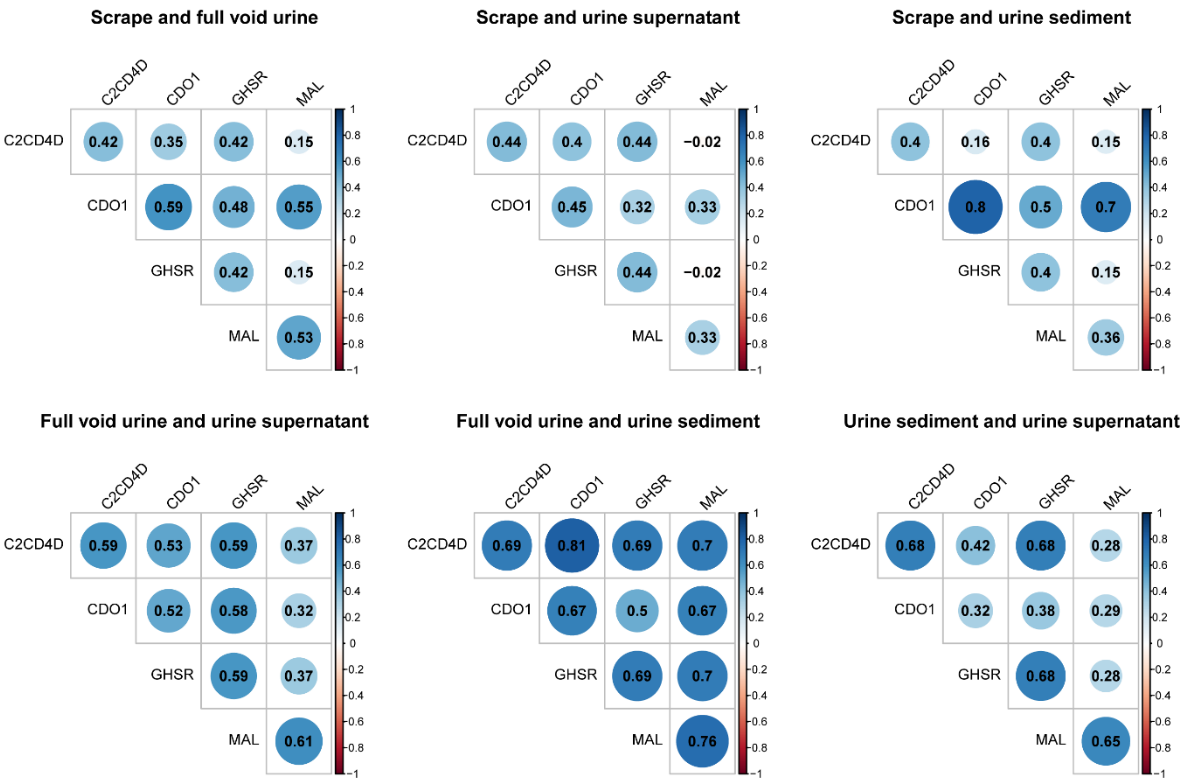

Supplemental Figure 10: Genome-wide SCNA profiles of matched urine and FFPE primary tumor tissue The log2 tumor to normal ratio is depicted on the y-axis and the chromosomal position on the x-axis. Computed using ichorCNA software. FFPE = formalin-fixed paraffin-embedded, SCNA = somatic copy number aberrations. Created with BioRender.com.

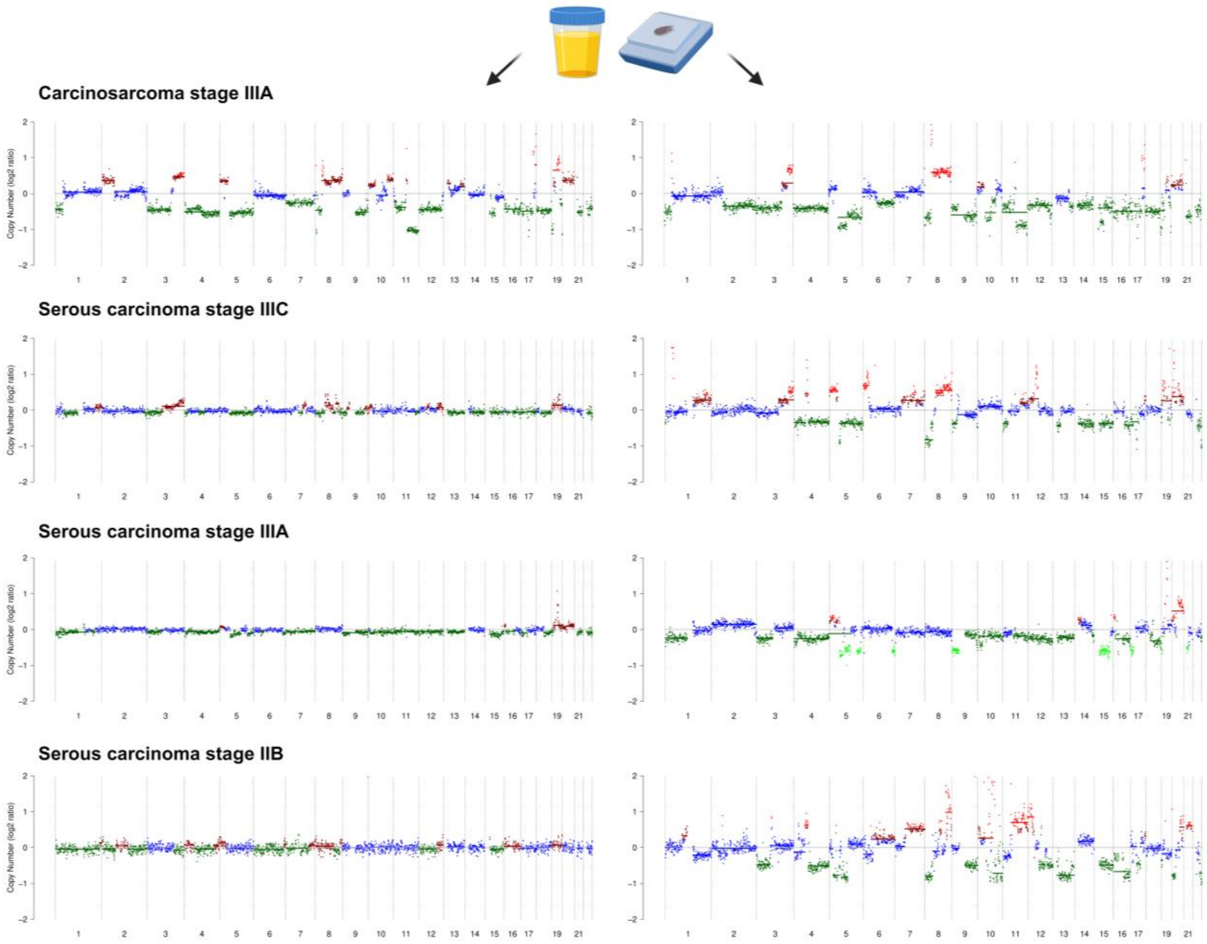

Supplemental Figure 11: Scatter plot indicating the relation between *MAL* methylation levels and the tumor fraction as estimated by ichorCNA in urine supernatant samples. *MAL* methylation levels are shown by 2log-transformed Cq ratios. *MAL* was the most discriminating marker between urine supernatant samples of healthy controls and ovarian cancer patient and therefore plotted against the tumor fraction. The patient with the highest tumor fraction in urinary cfDNA also showed the highest *MAL* methylation, as seen in the upper right part of the plot. Cq = quantification cycle.

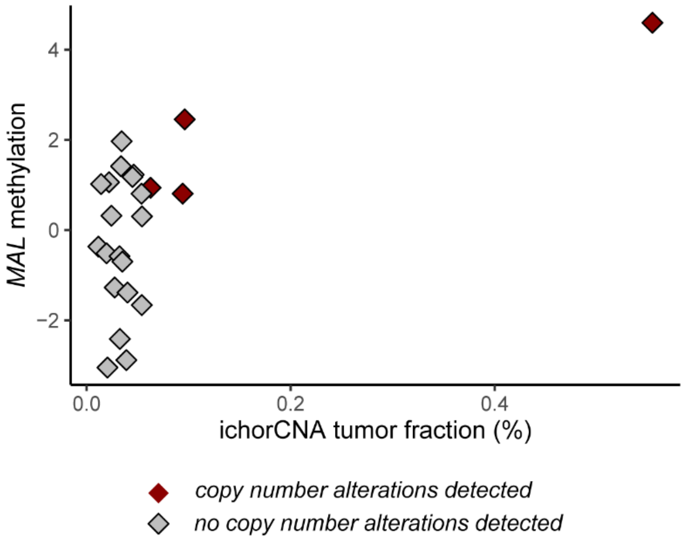

Supplemental Figure 12: Fragment size distributions for cfDNA reads of urine supernatant samples from healthy controls (n=2) and ovarian cancer patients with a low (<5%, n=19) and high (≥5%, n=4) tumor fraction determined from shallow whole-genome sequencing. The cfDNA with a high tumor fraction revealed a shorter modal fragment size (80 bp) than cfDNA with a low tumor fraction and controls (111 bp).

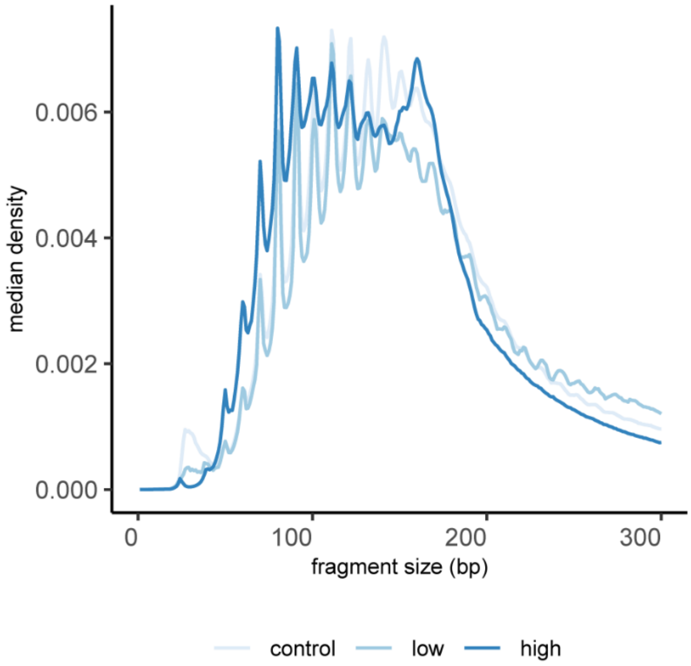

SUPPLEMENTAL TABLES

Supplemental Table 1: Primer and probe information of multiplex quantitative methylation-specific PCR assays.

**Multiplex**	**Target**	**Forward primer**	**Reverse primer**	**Probe**	**5'Dye-3'Quencher**	**Amplicon size**	**Amplicon location**	**Tm (°C)**
1	*GHSR*	available upon request	available upon request	available upon request	FAM - BHQ1	88	available upon request	63
1	*SST*	GCGTTGGTTGCGTTGTTTATC	CTACAAAAACTAACGAAATCTAAAATCCG	CCGATAACACAACCCAAAA	VIC - MGB-Eclipse®	84	Chr3: 187670184-187670267	63
1	*ZIC1*	available upon request	available upon request	available upon request	DFO - BHQ2	84	available upon request	63
2	*CDO1*	CGTTTTTTTTCGTTTTATTTTCGTCG	CCTCCGACCCTTTTTATCTACG	TGTGGTTCGCGACGTTGGGACGT	FAM - BHQ1	69	Chr5: 115816927-115816995	60
2	*MAL*	CGCGTAGTATTAAGTAGAGAGGTTCG	ACCGCCGACCCCTTCC	CCACTAAACCGACGCTAATTCGACGCT	DFO - BHQ2	85	Chr2: 95025113-95025197	60
2	*PRDM14*	TTACGTGTTATTGTCGGGGATTC	ATATCTATTCCTAATACCTAAAAACGAAACG	AAACGCCTTAAACGCTAAAAAACTTCGCCTC	JOE - BHQ2	88	Chr8: 70071718-70071805	60
3	*C2CD4D*	CGTGGGTCGTAGTTGGTAGTATAG	AACCCGCACTCGCCG	CGCCGAACCGCCC	DFO - MGB-Eclipse®	84	Chr1: 151838369-151838452	59
3	*GALR1*	available upon request	available upon request	available upon request	FAM - BHQ1	65	available upon request	59
3	*NRN1*	available upon request	available upon request	available upon request	JOE - BHQ1	65	available upon request	59
1,2	*ACTB*	available upon request	available upon request	available upon request	CY5 - MGB-Eclipse®	68	available upon request	63, 60
3	*ACTB*	available upon request	available upon request	available upon request	CY5 - MGB-Eclipse®	108	available upon request	59

Amplicon locations are based on GRch 38.109. Sequences not provided are available upon reasonable request from Self-screen B.V.

Supplemental Table 2: Analytical validation of multiplex quantitative methylation-specific PCR assays.

**Multiplex**	**Target**	**Slope**	**R2**	**Efficiency (%)**
1	*GHSR*	-3,38	1,00	97,52
1	*SST*	-3,24	0,99	103,69
1	*ZIC1*	-3,23	0,99	104,00
1	*ACTB*	-3,39	0,99	97,26
2	*CDO1*	-3,21	0,99	104,78
2	*MAL*	-3,28	0,98	101,89
2	*PRDM14*	-3,37	0,99	98,15
2	*ACTB*	-3,38	0,99	102,39
3	*C2CD4D*	-3,46	0,98	94,56
3	*GALR1*	-3,27	0,99	102,03
3	*NRN1*	-3,36	0,99	98,64
3	*ACTB*	-3,38	0,99	97,76

Data is based on serial dilution series of bisulfite treated methylated DNA from the SiHa cell line (100, 50, 10, 5, 1, 0.5%) within the range of 20 to 0.1 ng.
