# Supplementary material for: Molecular analysis for ovarian cancer detection in patient-friendly samples
Source: Commun Med (Lond). 2024 May 16;4:88. doi: 10.1038/s43856-024-00517-8 (PMC11099128; doi:10.1038/s43856-024-00517-8)
Supplement: Supplementary file 5 — Reporting Summary [file 43856_2024_517_MOESM5_ESM.pdf]

nature portfolio

Corresponding author(s): Renske D.M. Steenbergen

Last updated by author(s): Jan 31, 2024

Reporting Summary

Nature Portfolio wishes to improve the reproducibility of the work that we publish. This form provides structure for consistency and transparency in reporting. For further information on Nature Portfolio policies, see our Editorial Policies and the Editorial Policy Checklist.

Statistics

For all statistical analyses, confirm that the following items are present in the figure legend, table legend, main text, or Methods section.

n/a	Confirmed	
☐	☒	The exact sample size (*n*) for each experimental group/condition, given as a discrete number and unit of measurement
☐	☒	A statement on whether measurements were taken from distinct samples or whether the same sample was measured repeatedly
☐	☒	The statistical test(s) used AND whether they are one- or two-sided<br>*Only common tests should be described solely by name; describe more complex techniques in the Methods section.*
☐	☒	A description of all covariates tested
☐	☒	A description of any assumptions or corrections, such as tests of normality and adjustment for multiple comparisons
☐	☒	A full description of the statistical parameters including central tendency (e.g. means) or other basic estimates (e.g. regression coefficient) AND variation (e.g. standard deviation) or associated estimates of uncertainty (e.g. confidence intervals)
☒	☐	For null hypothesis testing, the test statistic (e.g. *F*, *t*, *r*) with confidence intervals, effect sizes, degrees of freedom and *P* value noted<br>*Give P values as exact values whenever suitable.*
☒	☐	For Bayesian analysis, information on the choice of priors and Markov chain Monte Carlo settings
☒	☐	For hierarchical and complex designs, identification of the appropriate level for tests and full reporting of outcomes
☐	☒	Estimates of effect sizes (e.g. Cohen's *d*, Pearson's *r*), indicating how they were calculated

Our web collection on statistics for biologists contains articles on many of the points above.

Software and code

Policy information about availability of computer code

Data collection

Data was collected using Castor EDC.

Data analysis

Methylation data was analyzed using R (version 4.0.3 with packages: cowplot, corrplot, dplyr, ggplot, ggpubr, and rstatix).

Processing of the sequencing data was performed by a pipeline controlled by Snakemake (v. 7.14.0). In brief, sequencing adapters and indexes were trimmed by the bbduk.sh (v. 38.79) [https://sourceforge.net/projects/bbmap/] in paired mode with parameters 'ktrim=r k=23 mink=11 hdist=1' and the adapter reference dataset provided with the software. Trimmed non-converted samples were mapped to the GRCh38 human genome assembly (GeneBank accession: GCA_000001405.28) using bwa mem (v. 0.7.17) [https://github.com/lh3/bwa]. Enzymatically converted reads were mapped to the same assembly using biscuit (v. 1.0.2.20220113) [https://huishenlab.github.io/biscuit/]. For both non-converted and converted samples, reads with a mapping quality lower than 5, unmapped reads, secondary mappings, chimeric and PCR duplicates were filtered using samtools (v. 1.12) [https://github.com/samtools/samtools] and sambamba markdup (v. 0.8.1) [https://lomereiter.github.io/sambamba/]. Reads passing the filtering step were submitted for somatic copy number aberrations (SCNA) analysis and tumor fraction estimation using the ichorCNA software (v. 0.3.2.0)7 using default settings, except the use of an in-house panel-of-normals from shallow whole-genome sequencing, setting the non-tumor fraction parameter restart values to c(0.95,0.99,0.995,0.999). The tumor fraction with the highest log likelihood was reported. Fragmentation patterns of urine cfDNA for both non-converted and converted samples were analyzed by retrieving the fragment sizes of the trimmed and filtered reads using picard CollectInsertSizeMetrics (v. 2.22.2) with HISTOGRAM_WIDTH=1000 [https://gatk.broadinstitute.org/hc/en-us].

Shallow whole-genome sequencing for the analysis of SCNA in paired FFPE primary tumor tissue was performed as described previously with a few adaptations8. Sequencing libraries were prepared using the KAPA HyperPlus Kit (Roche, Basel, Switzerland), following manufacturer's protocol. Libraries were sequenced using a NextSeq2000 (Illumina). Sequence reads were aligned to the GRCh38 human genome assembly

using bwa mem (v. 0.7.17). PCR duplicates (marked by Picard v. 2.20.8), as well as low-quality reads (MAPQ < 37), were filtered out using samtools (v. 0.1.1830). Reads passing the filtering step were submitted for SCNA analysis using ichorCNA software as described for urine samples.

For manuscripts utilizing custom algorithms or software that are central to the research but not yet described in published literature, software must be made available to editors and reviewers. We strongly encourage code deposition in a community repository (e.g. GitHub). See the Nature Portfolio guidelines for submitting code & software for further information.

Data

Policy information about availability of data

All manuscripts must include a data availability statement. This statement should provide the following information, where applicable:

- Accession codes, unique identifiers, or web links for publicly available datasets
- A description of any restrictions on data availability
- For clinical datasets or third party data, please ensure that the statement adheres to our policy

The sequencing dataset generated and analyzed during the current study is available in the European Genome-Phenome Archive repository, under accession number EGAD00001010848. The DNA methylation dataset generated and analyzed during this study is available from the corresponding author on reasonable request.

Human research participants

Policy information about studies involving human research participants and Sex and Gender in Research.

Reporting on sex and gender

Findings only apply to the female sex due to the characteristics of the studied cancer (ovarian cancer).

Population characteristics

A flowchart describing the study overview and sample types used is shown in Figure 1. Tissue samples of normal fallopian tube (n=22) and HGSOC (n=35) were collected to verify the discriminatory power of qMSP assays. Patient-friendly samples (urines, cervicovaginal self-samples, and clinician-taken cervical scrapes) were prospectively collected from 54 patients undergoing pelvic surgery at a tertiary oncology center because of a highly suspicious ovarian mass. Twenty-nine women were diagnosed with ovarian cancer and 25 with a benign ovarian mass. For comparison, 110 non-paired samples of healthy age-matched controls were collected from different settings, including 30 urines, 40 cervicovaginal self-samples and 40 clinician-taken cervical scrapes. Clinical characteristics of study participants of which patient-friendly material was collected are summarized in Table 1.

Recruitment

This study prospectively included patients with a highly suspicious ovarian mass according to current triage methods (>40% risk of malignancy using the IOTA adnex model)29,30. Paired samples (i.e., urine, cervicovaginal self-samples, and clinician-taken cervical scrapes) were consecutively collected within the SOLUTION1 study, between July 2018 and September 2022, at the Antoni van Leeuwenhoek hospital, Amsterdam, The Netherlands. Samples were collected from patients who underwent pelvic surgery with post-operatively confirmed ovarian cancer of any stage and histological subtype, and patients with a benign ovarian mass who were referred to a highly specialized tertiary oncology unit for further assessment. Patients scheduled for pelvic surgery, involving exploratory laparotomy to determine the origin of their ovarian mass or cytoreductive surgery, were asked to collect samples prior to surgery. Patients who could not collect cytological or urine samples prior to surgery were excluded from participation. Patients diagnosed with a borderline tumor were also excluded to focus on the most distinct tumor types in this exploratory stage (i.e., benign and malignant ovarian masses). Patients were included in the study regardless of whether all three paired sample types were available or not. For example, if a cervical scrape was not collected, the urine and self-sample of this patient were still analyzed and included.
Control urine samples were obtained from the URIC biobank, including healthy women without any prior cancer diagnosis within the last five years. Control cervicovaginal self-samples and cervical scrapes were retrieved from leftover material of the Dutch national cervical cancer screening program. Healthy control samples were within the same age range as women diagnosed with an ovarian mass and all tested negative for high-risk human papillomavirus (HPV). Information on prior benign gynecological disease and menopausal status was not documented for healthy control women. Yet, the majority of women were most likely postmenopausal with 93% of healthy control women aged over 50 years.

Ethics oversight

All patients participating in the SOLUTION1 study were 18 years or older and signed informed consent before sample collection. Ethical approval was obtained by the Medical Ethical Committee of the VU University Medical Center for the use of samples collected within the SOLUTION1 study (METc: 2016.213, Trial registration ID: NL56664.029.16), samples stored in the URIC biobank (TcB 2018.657), and samples archived in the biobank containing leftover material of the Dutch national cervical cancer screening program (TcB 2020.245). The Code of Conduct for Responsible Use of Left-over Material of the Dutch Federation of Biomedical Scientific Societies was adhered for the use of tissue specimen.

Note that full information on the approval of the study protocol must also be provided in the manuscript.

Field-specific reporting

Please select the one below that is the best fit for your research. If you are not sure, read the appropriate sections before making your selection.

☒ Life sciences ☐ Behavioural & social sciences ☐ Ecological, evolutionary & environmental sciences

For a reference copy of the document with all sections, see nature.com/documents/nr-reporting-summary-flat.pdf

Life sciences study design

All studies must disclose on these points even when the disclosure is negative.

Sample size	A total of 428 samples of 164 participants were analyzed within this study. No power calculations have been performed as this study is still in the feasibility stage. No previous data exists on ovarian cancer detection in urine and self-collected cervicovaginal samples by DNA methylation or copy number analysis. Limited data on ovarian cancer detection by DNA methylation analysis included 30 ovarian cancer patients and 30 controls which was sufficient to find a statistically significant difference (PMID: 29185275).
Data exclusions	Patients who could not collect cytological or urine samples prior to surgery were excluded from participation. Patients diagnosed with a borderline tumor were also excluded to focus on the most distinct tumor types in this feasibility stage (i.e., benign and malignant ovarian masses). Sample quality and sufficient input was ensured by excluding samples with a ACTB quantification cycle (Cq) ≥ 32.
Replication	Reproducibility of methylation measurements were verified by the careful design and optimization of qMSP assays before testing clinical samples of which limited amounts of (cf)DNA is available. All qMSP assays were designed, multiplexed and optimized according to parameters described earlier (27). Target specificity was validated in silico (BLAST). Correct amplicon size was verified by agarose gel electrophoresis. Analytical validation was performed using a dilution series of bisulfite treated methylated DNA from the SiHa cell line (100, 50, 10, 5, 1, 0.5%) within the range of 20 to 0.1 ng (Supplemental Table 2). The discriminatory power of each assay was verified by comparing methylation marker levels in tissue samples of ovarian cancer patients with those measured in normal fallopian tube tissue.
Randomization	Not relevant to our study. All groups were subjected to the same molecular analysis and statistical comparisons were made per diagnostic category (e.g., healthy control, benign ovarian mass, malignant ovarian mass).
Blinding	This study prospectively included patients with a highly suspicious ovarian mass according to current triage methods. Lab personnel was blinded to diagnosis (i.e., benign or malignant ovarian mass) during sample processing and methylation marker testing. Only urine samples of women diagnosed with ovarian cancer were sequenced, therefore, blinding was not possible for sequencing.

Reporting for specific materials, systems and methods

We require information from authors about some types of materials, experimental systems and methods used in many studies. Here, indicate whether each material, system or method listed is relevant to your study. If you are not sure if a list item applies to your research, read the appropriate section before selecting a response.

Materials & experimental systems

n/a	Involved in the study	
☒	☐	Antibodies
☒	☐	Eukaryotic cell lines
☒	☐	Palaeontology and archaeology
☒	☐	Animals and other organisms
☐	☒	Clinical data
☒	☐	Dual use research of concern

Methods

n/a	Involved in the study	
☒	☐	ChIP-seq
☒	☐	Flow cytometry
☒	☐	MRI-based neuroimaging

Clinical data

Policy information about clinical studies

All manuscripts should comply with the ICMJE guidelines for publication of clinical research and a completed CONSORT checklist must be included with all submissions.

Clinical trial registration	The current study includes clinical data but is not a clinical trial.
Study protocol	The current study includes clinical data but is not a clinical trial.
Data collection	The current study includes clinical data but is not a clinical trial.
Outcomes	The current study includes clinical data but is not a clinical trial.
