# Supplementary material for: Molecular analysis for ovarian cancer detection in patient-friendly samples
Source: Commun Med (Lond). 2024 May 16;4:88. doi: 10.1038/s43856-024-00517-8 (PMC11099128; doi:10.1038/s43856-024-00517-8)
Supplement: Supplementary file 6 — Description of Additional Supplementary Files [file 43856_2024_517_MOESM6_ESM.pdf]

Description of Additional Supplementary Files

File name: Supplementary Data 1

File Description: Shallow whole-genome sequencing coverage and quality statistics per sample.
